# How to publish a book

**DOI:** 10.1097/IJ9.0000000000000026

**Published:** 2017-06-15

**Authors:** Mohammad Al-Ubaydli, Katharine Whitehurst, Kiron Koshy, Buket Gundogan, Riaz Agha

**Affiliations:** a University College London; b Royal Devon and Exeter Hospital; c Brighton and Sussex University Hospitals; d University College London Medical School; e Guy's and St Thomas' NHS Foundation Trust, London, UK

**Keywords:** How to, Publish, Book, Publishing

## Abstract

Writing a book can seem like a daunting prospect. However, with the right idea, motivation, and effort, it is entirely possible. In this “How to” article we demonstrate and explain how one can take their book idea and successfully deliver it to publication.

In this article, we inform readers on the topic of book publishing and how to go about it. Book publishing has taken a back seat with the rise of online information that is readily updated and accessible. Books themselves have evolved with the advent of ebooks and devices like Kindle, IPAD, and mobile phones with larger more high-quality screens. However, many of the principles of book publishing remain the same. In this article, the authors give insights based on their experiences as readers, authors, and editors.

## Why write a book?

Writing your own book may seem daunting, but with the right idea, motivation, and effort, it is possible. Writing a book looks good on your CV, provides a small supplementary income, and opens doors to other writing opportunities.

Writing and publishing a book takes several steps:

1. The idea
2. Planning the book
3. Writing the book
4. Publishing the book

## Step 1: the idea

There are many medical books out there already. You need to identify a gap in the market.

Consider the following points:

- What is your book’s unique selling point?
- Research what’s already out there—how is your book different? What is good about them, and what can your book add?
- Are you the right person to write this book? Eg, A medical student writing on how to pass the MRCS is not appropriate.
- Who is the target audience? Define them and how you would get to them. Conduct market research among the target audience—is this something they’d be interested in? Is there anything they particularly want to be included?

## Step 2: planning the book

Moving from an idea to a plan is a vital step for success.

- What type of book is it? Eg, careers advice/revision/textbook/practice questions.
- Who is your target audience?—Level, specialty, country.
- Scope—which things will your book cover, and which things will it not.
- Authors—will it be written alone/as a group? Do you need to enlist help?
- Creating an outline—creating a mock contents page can be useful.
- Identify a mentor—Is there somebody who has knowledge in this field that can help you? It is best if they have experience in publishing, especially with a particular publisher you’re aiming for.

## Step 3: writing the book

It is important to ensure the idea, research, and planning have been thoroughly considered before moving onto writing. If you’re going to send it to a publisher, you will only need to write the first few chapters.

- Make a timetable for writing, ideally doing a bit everyday. Make realistic deadlines, and stick to them. Schedule breaks for when you have holidays/examinations/on-calls.
- Make use of trusted colleagues—ask them to review and proof read what you’ve written.

## Step 4: publishing the book

There are two (not necessarily mutually independent) ways to publish a book:

- Online
- In print

### Publishing a book online

If you don’t want to publish through an established publishing house, you can publish your work independently, for example through Amazon’s Kindle Direct Publishing.

PROS—Easy, quick, reaches a wide audience, more autonomy.

CONS—Less kudos, not reviewed.

### Publishing in print

For many people, this may be the most daunting part. Having done the earlier planning stages will help you write a good book proposal. Try not to be demoralized if your proposal is rejected, there are plenty of publishers out there. If you receive several rejections you’ll have to consider if your book is really needed.

The process to publishing your book will include the following stages:

- Select a publisher appropriate for your book.
- Write a robust book proposal, this should include:
  - A synopsis of your book.
  - The target audience.
  - The unique selling point.
  - The likely length of the book.
  - A list of complementing and competing books.
  - A few sample chapters.
  - Some publishers will also stipulate other details—please ensure you check.
- If accepted, you’ll sign a contract—know your rights.
- If rejected, submit to another publisher.
- Stick to agreed deadlines—contracts may become void if they are breached.
- Pay attention to feedback.
- Once you’ve completed a final draft, there are several steps before your book is printed:
  - Submit final draft to publisher.
  - Peer review.
  - Draft accepted.
  - Publisher typesets manuscript.
  - Author sent proof from publisher, makes changes, then agrees final proof.
  - Indexing book.
  - Book published.

PROS—More status, support/advice/reviews given.

CONS—Takes longer, less autonomy.

## Useful links

Kindle Direct Publishing: https://kdp.amazon.com/edu. European Medical Writers Association: http://www.emwa.org/.

## Conclusions

Writing a book is a good way to boost your CV, and give you more opportunities in medical writing. Writing a book requires discipline and good planning is essential. Books can be published through publishing houses, or increasingly self-published online.

